# Potassium‐Impurity‐Modulated Self‐Trapped Excitons and Energy Transfer in Phase‐Separated, Mn^4+^‐Doped Li_2_HfF_6_

**DOI:** 10.1002/advs.78338

**Published:** 2026-10-27

**Authors:** Menuka Adhikari, Thomas Murray, Cameron J. Lewis, Jett Wu, Hari Adhikari, Cheng Li, Jianguo Wen, Hui Wu, Zhiping Luo

**Affiliations:** ^1^ Department of Chemistry, Physics, and Materials Science Fayetteville State University Fayetteville North Carolina USA; ^2^ Center for Nanoscale Materials Argonne National Laboratory Lemont Illinois USA; ^3^ Neutron Scattering Division Oak Ridge National Laboratory Oak Ridge Tennessee USA; ^4^ NIST Center for Neutron Research National Institute of Standards and Technology Gaithersburg Maryland USA

**Keywords:** density functional theory (DFT), doping, fluoride, impurity, Li_2_HfF_6_, luminescence, self‐trapped exciton

## Abstract

Self‐trapped excitons (STEs) play an important role in the luminescence of halide materials, yet their formation and manipulation in complex fluorides remain largely underexplored. Here, Mn^4+^‐doped Li_2_HfF_6_ phosphors were synthesized by room‐temperature coprecipitation to investigate impurity‐modulated STEs and their role in Mn^4+^ sensitization. High‐resolution neutron powder diffraction reveals impurity‐associated phase separation into two closely related Li_2_HfF_6_ lattices. Photoluminescence and cathodoluminescence measurements resolve two broad host‐emission components: STE1, assigned to intrinsic Li_2_HfF_6_, and redshifted STE2, associated with a K‐containing lattice. Under high‐energy cathodoluminescence excitation, enhanced Mn^4+^ red emission correlates with STE2 emission but not with STE1 emission, supporting a selective STE2 → Mn^4+^ energy‐transfer pathway. Density functional theory ΔSCF (self‐consistent field) calculations yield distinct relaxed STE1 and STE2 configurations, confirming the K‐induced redshift of the STE emission. These results identify K‐associated STE2 as a key sensitizing state for Mn^4+^ luminescence, establishing impurity modulation as an effective strategy for tuning excitonic emission and host‐to‐activator energy transfer in complex fluoride phosphors.

## Graphical abstract

Li_2_HfF_6_ contains two spectroscopically distinct self‐trapped excitons (STEs): intrinsic STE1 and redshifted, K‐associated STE2. Potassium incorporation stabilizes STE2, which selectively sensitizes Mn^4+^ red emission. This work uncovers the often‐overlooked role of trace K impurities, demonstrating impurity modulation as an effective strategy for tuning excitonic emission and host‐to‐activator energy transfer in complex fluorides.

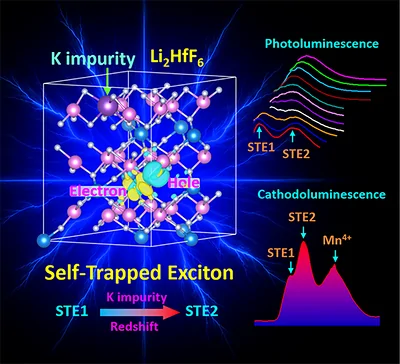

## 1 Introduction

Self‐trapped excitons (STEs) form when strong exciton–phonon coupling produces a local lattice distortion in a soft host, causing a localized excited electron–hole pair. Subsequent radiative decay of the relaxed STE produces a broad emission band with a large Stokes shift [1, 2]. In doped metal halides, STEs can transfer excitation energy to activator ions. STE‐mediated energy transfer has been demonstrated for transition‐metal and rare‐earth (RE) dopants, including Mn^2+^ [3, 4, 5], Er^3+^ [6], Ho^3+^ [7], Tb^3+^ [8], and Nd^3+^ [9], as well as for ns^2^ metal ions such as Sb^3+^ [10]. In these systems, STEs provide an indirect broadband excitation pathway to the activator, enhancing dopant‐centered visible or near‐infrared emissions.

The STE can be tuned by modifying the composition and local structure of the host lattice. Ke et al. tuned the broad STE emission of Cs_2_Na_1 − *x*_Ag_*x*_BiCl_6_ by varying the Na/Ag composition [11]. Chang et al. showed that introducing a small amount of Rb^+^ into Te^4+^‐doped Cs_2_ZrCl_6_ enhanced photoluminescence (PL) intensity and moisture stability [12]. It was also found that Te^4+^ incorporation into Cs_2_ZrCl_6_ introduces a Te^4+^‐related excited state that undergoes strong lattice relaxation, producing broad yellow STE emission [13]. The Lin group doped the Cs_2_Na(Lu/Er)Cl_6_ double perovskite with Sb^3+^ at the RE‐ion site, significantly increasing the STE → Er^3+^ energy‐transfer efficiency [6]. In addition, Cu^+^ substitution in Cs_2_(Ag/Na)InCl_6_ enhances the host STE emission by breaking the local symmetry around the Ag^+^ site and increasing the radiative recombination rate of STEs [14], and Sb^3+^ incorporation can generate additional dopant‐associated STE in hybrid manganese chloride [15]. More recently, Li et al. obtained excitation‐dependent dual STE bands by Bi^3+^/Te^4+^ codoping of Rb_2_SnCl_6_, with asymmetric energy transfer between the two emissive states [16], while Wang et al. demonstrated parallel STE → Er^3+^ and Dy^3+^ → Er^3+^ energy‐transfer pathways in Cs_3_DyI_6_:Er^3+^ nanocrystals [17].

Although STEs have attracted significant attention in recent years, studies on STE luminescence and STE‐mediated energy transfer in metal halides have largely focused on chlorides, bromides, and iodides, particularly low‐dimensional and vacancy‐ordered halide perovskites as mentioned above [1, 2, 3, 4, 5, 6, 7, 8, 9, 10, 11, 12, 13, 14, 15, 16, 17]. In contrast, self‐trapping in fluorides has historically been investigated primarily in classical ionic crystals, such as alkali and alkaline‐earth fluorides (LiF, MgF_2_, CaF_2_, SrF_2_, BaF_2_), and simple fluoride perovskites (KMgF_3_, RbMgF_3_) [18]. More recently, broad STE emission and STE‐mediated energy transfer to RE activators have been demonstrated in the complex fluoride Cs_2_HfF_6_ [19]. Although fluoride compounds are attractive hosts because their relatively low maximum phonon energies can reduce multiphonon nonradiative relaxation and facilitate efficient radiative emission [20, 21], tuning their STE states and their use for sensitizing activator emission remain largely underexplored in complex fluoride systems.

The ternary fluoride Li_2_HfF_6_ crystallizes in the trigonal $P\bar{3}1m$ structure, where both Hf^4+^ and Li^+^ occupy octahedrally coordinated fluoride environments, which differs from the structure of Cs_2_HfF_6_ [22]. This unique structure provides a platform for investigating substitution‐controlled excitonic processes because the two cation sublattices can play distinct roles in luminescence. The octahedrally coordinated Hf^4+^ site provides a charge‐matched environment for activator substitution, while the Li‐containing sublattice accommodates a substantially larger alkaline ion, thereby introducing pronounced local structural distortion and modifying lattice relaxation around excited states. Additionally, the wide bandgap, highly ionic fluoride lattice provides a favorable environment for localized excitations and self‐trapping. Although the crystal structure of pure Li_2_HfF_6_ and, more recently, the PL of Mn^4+^‐doped Li_2_HfF_6_ have been reported [22, 23], the formation of STEs and their microscopic origins in this material have not been established.

In this work, Mn^4+^‐doped Li_2_HfF_6_ was synthesized by solution coprecipitation using K_2_MnF_6_ as the Mn^4+^ precursor. This precursor simultaneously introduces Mn^4^^+^ activators and K^+^ ions, enabling examination of the accompanying alkali‐metal impurity. An unusual phase separation gives rise to two distinct STE emissions: intrinsic STE1, associated with intrinsic Li_2_HfF_6_, and redshifted STE2, associated with K‐containing Li_2_HfF_6_. Through combined structural and compositional characterization, PL and CL (cathodoluminescence) spectroscopy, and first‐principles calculations, we demonstrate that K incorporation perturbs the Li_2_HfF_6_ lattice, thereby stabilizing a redshifted self‐trapped excited state. STE2 selectively sensitizes the characteristic red emission of Mn^4+^. These findings identify K^+^ impurities as significant yet often overlooked contributors to the optical response, demonstrating impurity‐modulated self‐trapping as a strategy for tuning host‐to‐activator energy transfer in fluoride‐based luminescent materials.

## 2 Experimental and Computational Methods

### 2.1 Materials Synthesis

LiF (98.5%) was purchased from Beantown Chemical (Hudson, New Hampshire, USA), and HfCl_4_ (99.9%) was purchased from Strem Chemicals (Newburyport, Massachusetts, USA). KMnO_4_ (99%) and KF (99%) were obtained from Alfa Aesar, and H_2_O_2_ (30%) and HF (40 vol.%) were obtained from Sigma–Aldrich. All were used as received.

K_2_MnF_6_ was prepared using a modified version of the reported fluoride‐precipitation procedure [24]. Briefly, KF (6.7 g) was dissolved in 30 mL of 40% HF in an ice bath. KMnO_4_ (0.45 g) was then added with stirring until fully dissolved, followed by the slow, dropwise addition of 0.4 mL of 35% H_2_O_2_. The solution changed from deep brown to light brown, and a yellow precipitate formed. The mixture was removed from the ice bath, and the precipitate was collected by centrifugation and washed several times with acetone and ethanol. In the final washing step, 1 mL of 40% HF was added to improve purity. The powder was dried at 70 °C overnight.

Li_2_HfF_6_ and Mn^4^^+^‐doped Li_2_HfF_6_ were prepared by a two‐step coprecipitation route. LiF (2 mmol) was mixed with 1 mL of 6 m HCl, stirred for 10 min, heated at 60°C for 15 min, and then stirred for an additional 30 min after the heat source was removed. Deionized water (1 mL) was added. Separately, 1 mL of 1 m aqueous HfCl_4_ was mixed with 1 mL of 40% HF and immediately added to the Li‐containing solution. The mixture was poured into approximately 20 mL of ethanol to precipitate Li_2_HfF_6_. The product was collected by centrifugation, washed three times with ethanol with sonication between washes, and dried at 70 °C. For Mn^4+^‐doped samples, the desired amount of K_2_MnF_6_ was dissolved in 1 mL of 40% HF and mixed with the HfCl_4_ solution before precipitation. A K‐doped, Mn‐free reference sample was synthesized separately by replacing an appropriate amount of KF with LiF for comparison.

To fabricate a phosphor‐converted light‐emitting diode (pc‐LED), 95.4 mg of Mn^4+^‐doped Li_2_HfF_6_ was mixed with Y_3_Al_5_O_12_:Ce^3+^ (YAG:Ce^3+^) powder at a mass ratio of 1:1. After thoroughly grinding the powders in an agate mortar, approximately an equal mass of epoxy polymer solution was added to form a composite. The resulting composite was then coated onto a blue LED chip for pc‐LED testing.

### 2.2 Structural Characterization and Luminescence Studies

X‐ray diffraction (XRD, Rigaku MiniFlex 600) was used to identify phases. High‐resolution neutron powder diffraction (NPD, HB‐2A, Oak Ridge National Laboratory) was used to improve sensitivity to the light‐element sublattice. Particle morphology and crystallinity were examined using a transmission electron microscope (TEM, Thermo Scientific Talos F200X) at 200 kV. Energy‐dispersive spectroscopy (EDS) mapping was performed in scanning TEM (STEM) mode using a high‐angle annular dark‐field (HAADF) detector. PL spectra were collected at room temperature using a Horiba FluoroMax Plus Steady State and Lifetime Benchtop Spectrofluorometer, and PL decay time was measured using a Horiba EasyLife L Phosphorescence Lifetime Spectrometer. CL spectra were acquired using a JEOL field‐emission JXA‐8530F electron probe microanalyzer (EPMA) equipped with an xCLent IV advanced hyperspectral CL detector, with accelerating voltages ranging from 5 to 30 kV.

### 2.3 Computational Methods

First‐principles calculations based on density functional theory (DFT) were carried out using the Vienna Ab initio Simulation Package (VASP) [25, 26], and periodic 2 × 2 × 3 supercells of trigonal Li_2_HfF_6_ were used. Core‐valence interactions were described using the projector‐augmented‐wave method [27], and exchange‐correlation effects were treated within the Perdew–Burke–Ernzerhof generalized‐gradient approximation [28]. A plane‐wave cutoff of 520 eV was used. The SCF convergence threshold was set at 1 × 10^−5^ eV, and structural relaxations were continued until the residual force on each atom was below 0.02 eV Å^−1^. An effective Hubbard parameter of *U*_eff_ = 12 eV was applied to the F 2p orbitals to stabilize the localized hole component. For the Mn 3d orbitals, *U*_eff_ = 4 eV was used, whereas no Hubbard correction was applied to the other elements. Spin polarization was performed for all calculations. The resulting spin‐density distributions were analyzed using VASPKIT [29].

## 3 Results and Discussion

### 3.1 Structural Characterization and Phase Separation

In the experimental materials, because Hf dominates the x‐ray scattering of Li_2_HfF_6_, high‐resolution neutron powder diffraction (NPD) was used to obtain greater sensitivity to the Li/F sublattice of nominally undoped Li_2_HfF_6_ (Figure 1a). The reflections are consistent with trigonal $P\bar{3}1m$ symmetry (No. 162). However, a careful Rietveld refinement of the NPD pattern reveals that two lattice sets are resolved, with structural parameters listed in Table 1. An enlargement of the NPD pattern is shown in Figure 1b, clearly indicating the presence of two sets of closely spaced Bragg reflections. The refinement yields a weighted profile residual *R*_wp_ = 3.1% and the goodness of fit GOF = 3.76.

**FIGURE 1:**
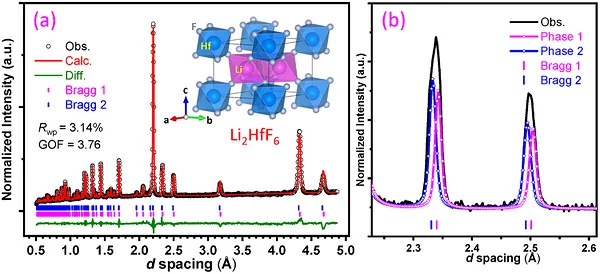
(a) High‐resolution NPD refinement of nominally undoped Li_2_HfF_6_, revealing contributions from two lattices; the inset shows the Li_2_HfF_6_ structural model, in which Hf and Li occupy the centers of F‐coordinated octahedra. (b) Enlarged diffraction region highlighting the contributions from the two lattices.

**TABLE 1:** Rietveld‐refinement results for the NPD pattern. Relative to Phase 1, Phase 2 exhibits lattice expansions of 0.37% along *a* and 0.40% along *c*. *R*_wp_ = 3.1%; GOF = 3.76.

Phase	1					2				
Lattice (Å)	*a* = 4.9844(1), *c* = 4.6610(2)					*a* = 5.0029(2), *c* = 4.6798(2)				
Fraction (wt.%)	54.0					46.0				
Atoms	*x*	*y*	*z*	Occ.	*B*	*x*	*y*	*z*	Occ.	*B*
Li	0.3333	0.6667	0.5000	1.00(4)	1.3(2)	0.3333	0.6667	0.5000	1.00(5)	2.0(4)
Hf	0.0000	0.0000	0.0000	1.00	0.20(5)	0.0000	0.0000	0.0000	1.00	1.44(10)
F	1.0000	0.3258(4)	0.2539(5)	1.00	0.59(3)	1.0000	0.3220(6)	0.2545(7)	1.00	1.10(5)

To investigate the origin of the phase separation, we analyzed the sample's chemical composition using EDS in EPMA. The EDS spectrum of nominally undoped Li_2_HfF_6_, collected after a prolonged 3 min acquisition, is shown in Figure S1a. Hf and F produce the major peaks, whereas weak K and Na signals become detectable after the long acquisition, as shown in the enlarged spectrum in Figure S1b. The C and O signals may arise from surface contamination or electron‐beam‐induced depositions during prolonged exposure. Quantitative analysis reveals approximately 0.1 at.% Na and 0.2 at.% K. If this alkali‐metal signal is treated as 0.3 at.% K only, since Li is not detectable by EDS, the measured composition is thus Li_1.98_K_0.02_HfF_6_, corresponding to a 1% substitution of K at Li sites. This value is within the uncertainty limits of the Rietveld refinement for the Li‐site occupancy by NPD. The apparent Cl, as indicated by its position in the spectrum, is only 0.01 at.%, which is within the uncertainty of the EDS measurement and was therefore not considered further. The trace alkali metals may originate from the starting reagent of the Li source. For comparison, we also synthesized a sample intentionally doped with 10% of K at the Li site, Li_2‐_ *_x_* K*_x_* HfF_6_ (*x* = 0.2). Its EDS spectrum is shown in Figure S1c, with an enlargement in Figure S1d. The K peak is evident, whereas Na and Cl remain unresolved. Quantification reveals 0.8 at.% K, corresponding to an estimated Li_1.94_K_0.06_HfF_6_ composition and only 3% substitution of Li sites. These results suggest that the slightly larger lattice parameters of Phase 2 may be due to alkali‐metal impurities. Substitution of larger alkali ions, Na^+^ (1.02 Å) and K^+^ (1.38 Å), for the smaller Li^+^ ion (0.76 Å) at Li sites may account for the observed lattice expansion.

DFT calculations were performed to quantify the relationship between the lattice expansion and K substitution. The fully relaxed Li_2_HfF_6_ unit cell has *a* = 4.848 Å and *c* = 4.534 Å. Supercells of several sizes were then constructed and fully relaxed, and one Li atom was replaced by K in each substituted model. The K substitution level is defined as the percentage of Li sites replaced by K, and the expansion is calculated by the fully relaxed K‐substituted supercell dimension compared with the corresponding supercell dimension without the substitution. Over the range investigated, the calculated expansion varies approximately linearly with the K substitution level, as shown in Figure 2 and Table S1, with the following relationship

(1) $$\varepsilon =0.2409x_{K},R^{2}=0.9989,$$

where *ε* is the lattice expansion in percent and *x*_K_ is the K substitution level in percent. The experimental expansions are 0.37% along *a* and 0.40% along *c* (Table 1), corresponding to K‐substitution levels of approximately 1.54% and 1.66%, respectively, according to Equation (1). The average estimated K substitution level is therefore approximately 1.6%. This value is slightly higher than 1% obtained by EDS. However, the EDS value represents an average over both phases. Assuming that all detected K is concentrated in Phase 2, which accounts for 46.0 wt.% of the sample, the local K substitution level in this phase would be 2.17%, reasonably close to the DFT‐based estimate.

**FIGURE 2:**
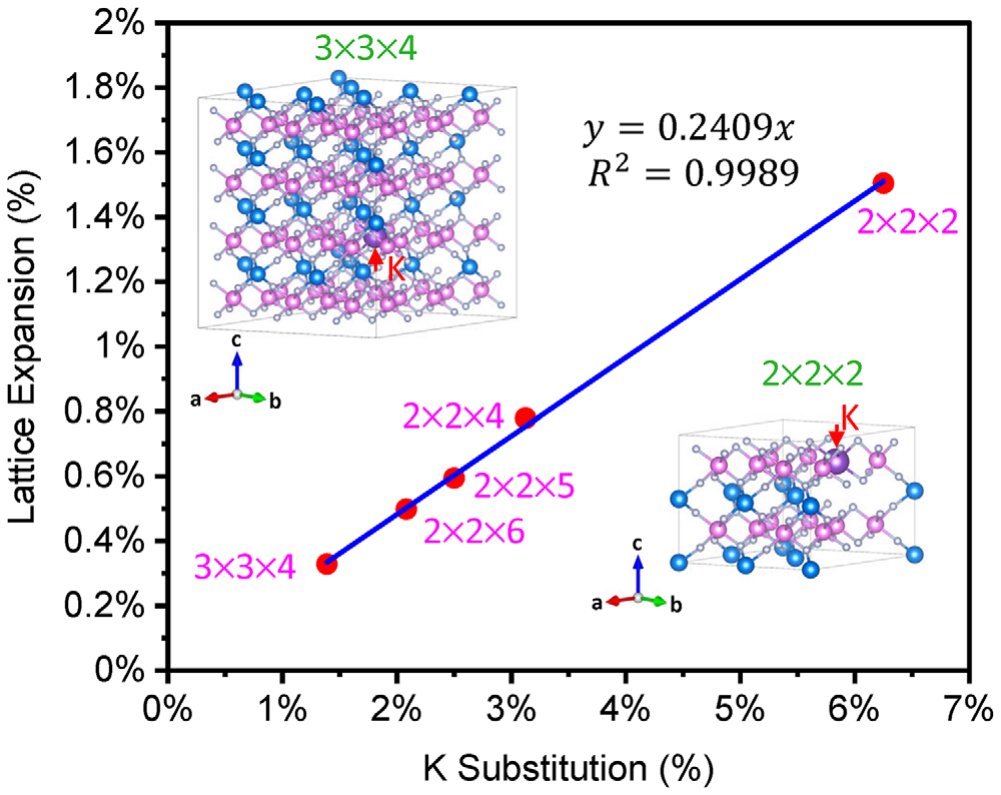
DFT‐calculated lattice expansion of Li_2_HfF_6_ as a function of K substitution level. Because the calculated expansions along the *a* and *c* directions are nearly identical, their average value is plotted. The supercell dimensions used for each substitution level are indicated next to the corresponding data points, and representative 2 × 2 × 2 and 3 × 3 × 4 supercells are shown in the insets.

As shown in Figure S2, the XRD patterns of Mn^4+^‐doped Li_2_HfF_6_ match the standard Li_2_HfF_6_ pattern (card No. 04‐020‐3207). No additional reflections are resolved for nominal Mn doping levels up to 3%, indicating that Mn^4+^ ions are incorporated into the host lattice by substituting for Hf^4+^. However, at Mn doping levels of 5% and higher, a weak impurity peak near 31° from KMnF_3_ appears, indicating the solubility limit and the onset of secondary‐phase formation by this synthesis method.

TEM reveals micrometer‐scale particles in the 2% Mn^4+^‐doped Li_2_HfF_6_ (Figure 3a). The EDS reveals the presence of the impurity K, which originated from the precursor K_2_MnF_6_, as shown in Figure 3b. The weak Ti signal is from the TEM system and is absent in the EPMA EDS. The selected‐area electron diffraction (SAED) pattern confirms the highly crystalline nature of the particles, as shown in the inset of Figure 3b. EDS mapping in STEM mode reveals uniform distributions of Hf, F, and Mn within the mapped region (Figure 3c–e), whereas K is distributed inhomogeneously (Figure 3f). This inhomogeneity is consistent with localized K‐rich environments that can perturb the Li sublattice.

**FIGURE 3:**
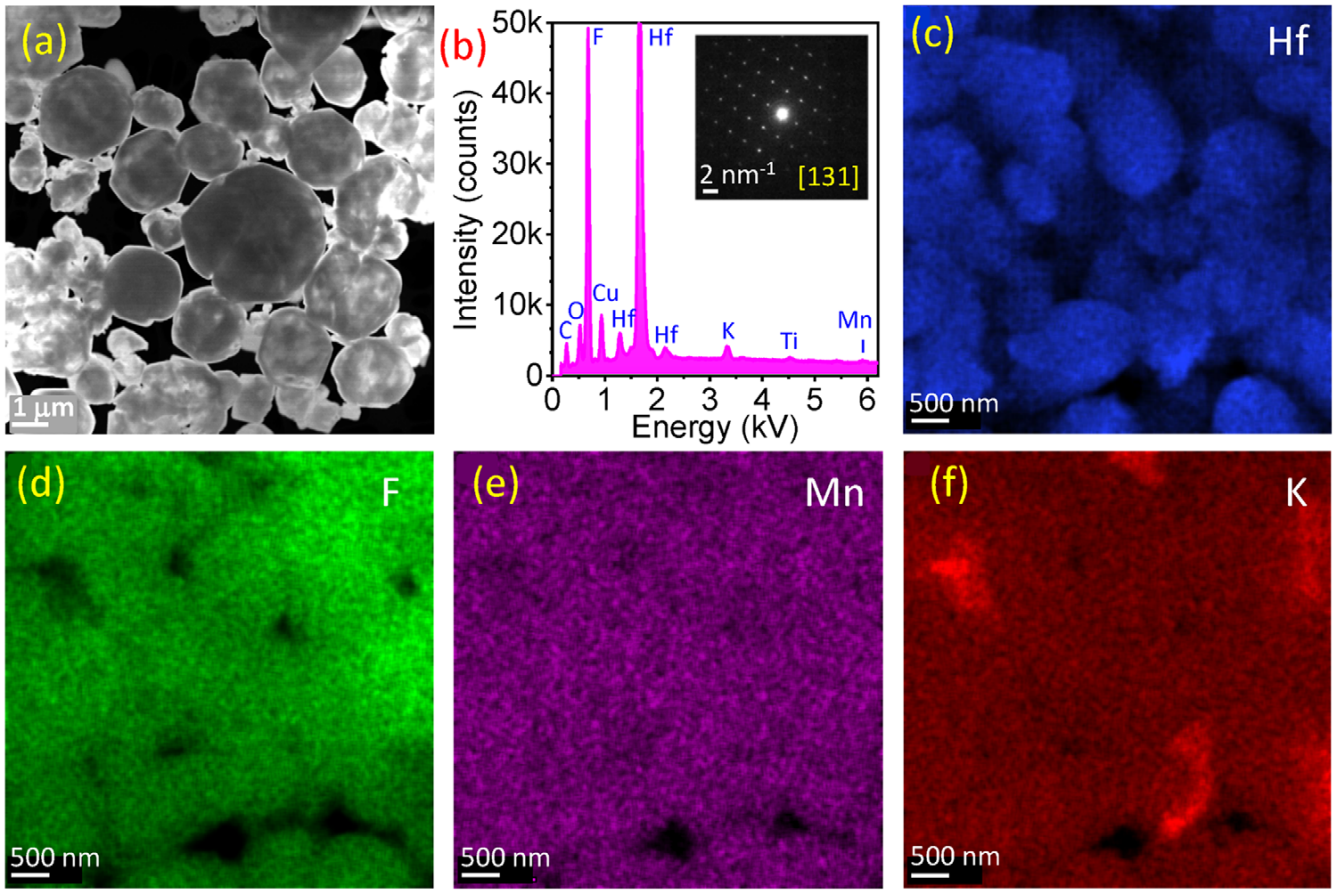
(a) TEM image of 2% Mn^4+^‐doped Li_2_HfF_6_ particles; (b) EDS showing the existence of K (inset is SAED pattern). (c–f) STEM‐EDS maps of Hf, F, Mn, and K, respectively. Hf, F, and Mn are uniformly distributed, whereas the K distribution is inhomogeneous.

During room‐temperature coprecipitation, rapid nucleation and precipitation may favor kinetically trapped lattice populations because long‐range cation redistribution and structural equilibration are limited. Trace alkali‐metal impurities may locally perturb the Li sublattice and promote the nucleation and growth of a slightly different Li_2_HfF_6_ lattice configuration, allowing coexisting lattices with different local K environments. Whether similar phase separation occurs in other structurally related compounds, such as Li_2_ZrF_6_, remains to be established.

### 3.2 Photoluminescence With Two Self‐Trapped Exciton Emissions

The excitation‐dependent PL spectra of nominally undoped Li_2_HfF_6_ are shown in Figure 4a. The spectra reveal two broad host‐emission channels whose relative contributions vary strongly with excitation wavelength. Under short‐wavelength excitation, a higher‐energy UV emission dominates, and its maximum shifts from 312 nm to approximately 415 nm (when excited at 360 nm), a change that cannot be clearly distinguished due to overlap with the next band. This emission is assigned to the intrinsic host STE and designated STE1. As the excitation wavelength increases, a lower‐energy blue emission progressively emerges, with maxima extending from approximately 405 to 467 nm, which is designated STE2. Because the nominally undoped sample contains only trace amounts of alkali‐metal impurities, STE2 is relatively weak under short‐wavelength excitation but becomes increasingly prominent at longer excitation wavelengths. Deconvolution of the spectrum excited at 360 nm (Figure 4b) resolves overlapping STE1 and STE2 components centered at 415.0 and 458.6 nm, respectively, confirming that both channels contribute in this excitation regime.

**FIGURE 4:**
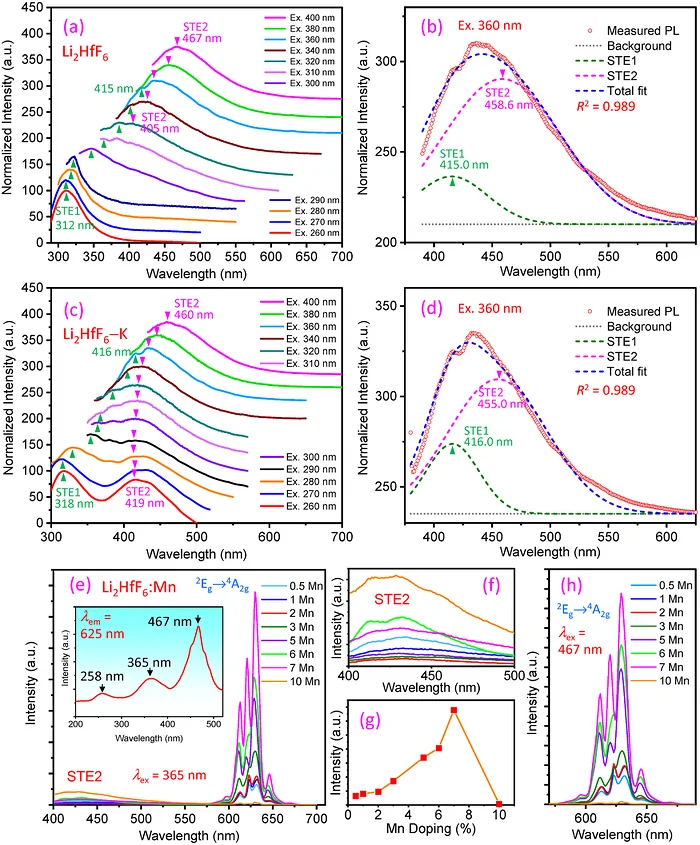
Photoluminescence. (a) Excitation‐dependent PL spectra of nominally undoped Li_2_HfF_6_, showing a higher‐energy STE1 emission under UV excitation and an increasingly dominant redshifted STE2 emission at longer excitation wavelengths. (b) Deconvolution of the PL spectrum under 360 nm excitation. (c) Excitation‐dependent PL spectra of intentionally K‐doped Li_2_HfF_6_, showing STE1 and STE2 contributions under UV excitation and enhanced STE2 emission with increasing excitation wavelength. (d) Deconvolution of the PL spectrum under 360 nm excitation. (e) PL spectra of Mn^4+^‐doped Li_2_HfF_6_ under 365 nm excitation, showing the broad STE2 emission together with the characteristic Mn^4+^ red emission; the inset shows the excitation spectrum monitored at 625 nm of the Mn^4+^ red emission, with excitation bands near 258, 365, and 467 nm. (f) Enlargement of the STE2 region in (e). (g) Intensity under 365 nm excitation as a function of Mn doping level. (h) Mn^4+^ red emission under direct 467 nm excitation.

In intentionally K‐doped Li_2_HfF_6_ (Figure 4c), STE2 is more pronounced over the entire excitation range. Under 260 nm excitation, emission features associated with STE1 and STE2 appear near 318 and 419 nm, respectively, while longer‐wavelength excitation increasingly favors the redshifted STE2 channel. Consistently, deconvolution of the 360 nm‐excited spectrum (Figure 4d) gives STE1 and STE2 components centered at 416.0 and 455.0 nm, respectively. A similar two‐component host emission is observed in the 10% Mn‐doped sample (Figure S3), in which STE2 remains particularly intense while the Mn^4+^ emission is strongly suppressed, consistent with concentration quenching and the increased formation of secondary phases at high Mn loading. The excitation‐dependent peak shifts likely reflect changes in the relative population of a distribution of local STE configurations rather than a rigid shift of a single relaxed excited‐state minimum. Different excitation wavelengths may preferentially access intrinsic, K‐perturbed, and defect‐associated local environments, thereby altering the apparent STE1 and STE2 peak positions and intensities.

Within the A_2_BF_6_‐type fluorides (A = Li, Na, K, Rb, or Cs; B = Hf, Zr, Ti, Si, Ge, or Sn in the 4^+^ state), most previous studies have focused on activator‐centered photoluminescence [23, 24, 30, 31, 32, 33, 34, 35, 36, 37, 38, 39, 40, 41, 42, 43], whereas host STE emission has received much less attention, with Cs_2_HfF_6_ representing a notable example [19]. The Mn^4+^‐doped Li_2_HfF_6_ samples with 0.5%, 1%, 2%, 3%, 5%, 6%, 7%, and 10% doping levels, denoted as 0.5Mn, 1Mn, 2Mn, 3Mn, 5Mn, 6Mn, 7Mn, and 10Mn, respectively, exhibit the characteristic Mn^4+^ red emission of the ^2^E_g_ → ^4^A_2g_ transition in the 600–650 nm region (Figure 4e). Under 365 nm excitation (Figure 4e), broad host STE emission and sharp Mn^4+^ emission are observed simultaneously. The excitation spectrum monitored at 625 nm of the Mn^4+^ red emission (inset of Figure 4e) shows three broad excitation bands near 258, 365, and 467 nm, assigned to the higher‐energy ^4^A_2g_ → ^4^T_1g_ (^4^P), second spin‐allowed ^4^A_2g_ → ^4^T_1g_ (^4^F), and first spin‐allowed ^4^A_2g_ → ^4^T_2g_ (^4^F) transitions, respectively [44, 45, 46]. The higher‐energy band was also assigned to the charge transfer between F^−^ and Mn^4+^ ions [47]. Because the broad STE emissions overlap these Mn^4+^ excitation bands, host → Mn^4+^ energy transfer is energetically feasible. In particular, the redshifted STE2 emission overlaps closely with the 467 nm Mn^4+^ excitation band. Figure 4f shows the enlargement of the STE region. For samples containing up to 7% Mn, STE2 emission is lower than that of the 10Mn sample, while Mn^4+^ emission is higher, consistent with an STE2 → Mn^4+^ energy‐transfer pathway. However, because Mn^4+^ is also directly excited at 365 nm, the PL spectra alone contain contributions from both direct Mn^4+^ excitation and host‐mediated sensitization. At 10% Mn, the Mn^4+^ emission decreases sharply due to concentration quenching and secondary phases, whereas its STE2 emission remains strong due to inefficient transfer from STE2 to Mn^4+^. The highest emission is recorded at 7% Mn, as shown in Figure 4g. Under 467 nm excitation (Figure 4h), Mn^4+^ is directly excited through its excitation band, producing the characteristic red emission.

Figure 5 compares the decay kinetics of the Mn^4+^ emission at 625 nm by ^2^E_g_ → ^4^A_2g_ transition under 260 and 467 nm excitation in a 3% Mn^4+^‐doped sample. Figure S4 shows the emission spectra under 260 nm excitation, revealing the STE2 emission that overlaps with the ^4^A_2g_ → ^4^T_2g_ (^4^F) band of Mn for energy transfer, while 467 nm directly excites Mn^4+^. Their decay profiles are well described by a biexponential function of intensity *I* with time *t*,

(2) $$I\left(t\right)=y_{0}+A_{1}e^{-t/\tau_{1}}+A_{2}e^{-t/\tau_{2}},$$

where *y*_0_ is a constant; *τ*_1_ and *τ*_2_ are fast and slow decay times; and *A*_1_ and *A*_2_ are two associated time‐invariant constants at *τ*_1_ and *τ*_2_, respectively. The average lifetime is calculated by

(3) $$\tau_{\mathrm{ave}}=\frac{A_{1}\tau_{1}^{2}+A_{2}\tau_{2}^{2}}{A_{1}\tau_{1}+A_{2}\tau_{2}}.$$

**FIGURE 5:**
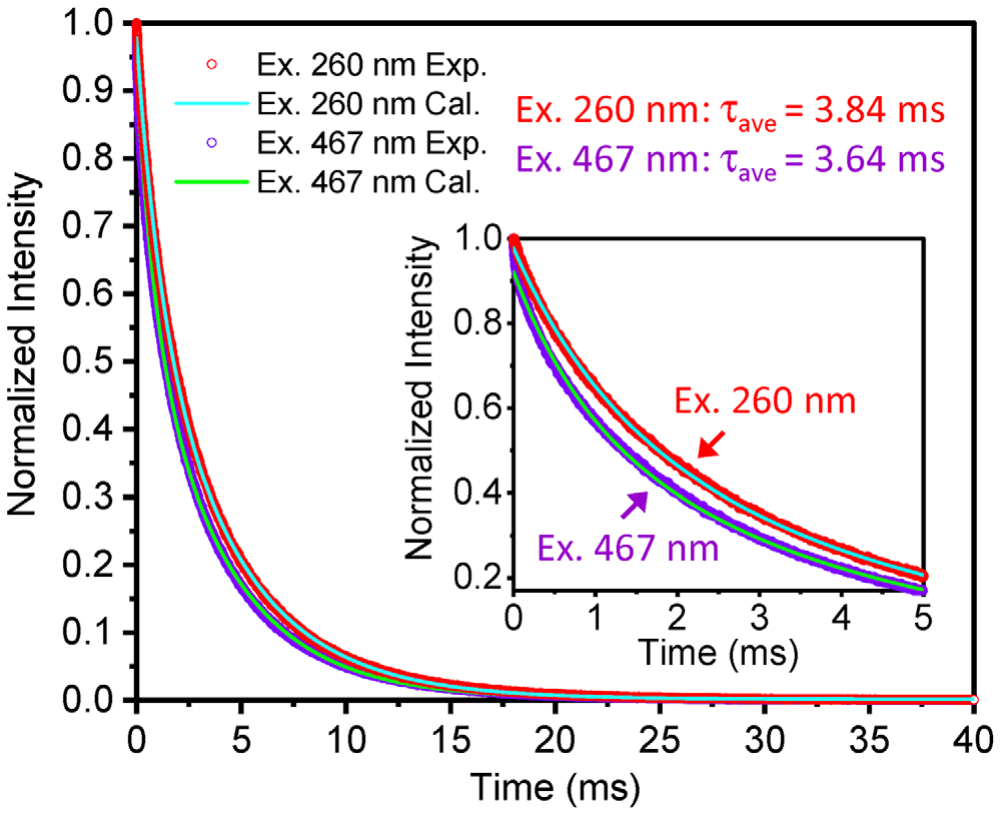
Time‐resolved decay curves of the Mn^4+^ emission (625 nm) under 260 and 467 nm excitation. The experimental decay profiles were fitted using a biexponential function. The inset enlarges the initial 0–5 ms region, highlighting the slightly slower decay under 260 nm excitation.

We conducted five measurements under 260 and 467 nm excitation, and the fitting parameters are listed in Table S2; the averaged fitting parameters are listed in Table 2. The fitting indicates the involvement of more than one relaxation component. The average lifetime increases from 3.64 ms under direct 467 nm excitation to 3.84 ms under 260 nm excitation, corresponding to an increase of approximately 5.5%. The measured lifetime is close to the recently reported result [23]. As shown more clearly in the enlarged early‐time region in the inset of Figure 5, emission excited at 260 nm decays systematically more slowly than emission excited at 467 nm. The longer lifetime under 260 nm excitation is consistent with an additional host‐mediated population pathway to the Mn^4+^ excited state, which delays Mn^4+^ emission. This observation therefore supports the proposed energy‐transfer process from the host excited states to Mn^4+^.

**TABLE 2:** PL decay fitting parameters of Figure 5.

Excitation (nm)	$\bar{y_{0}}$	$\bar{A_{1}}$	$\bar{\tau_{1}}$ (ms)	$\bar{A_{2}}$	$\bar{\tau_{2}}$ (ms)	τ_ave_ (ms)	Ave. Adj.‐*R* ^2^
260	0.00143	0.31968	1.00919	0.64803	4.17231	3.84 ± 0.03	0.99982
467	0.00114	0.32499	0.90078	0.60442	3.97261	3.64 ± 0.01	0.99971

To demonstrate the practical potential of Mn^4+^‐doped Li_2_HfF_6_ for solid‐state lighting, a pc‐LED was fabricated by coating Mn^4^^+^‐doped Li_2_HfF_6_ together with a yellow YAG phosphor onto a blue LED chip (Figure S5). The resulting device exhibits broad visible emission spanning the blue‐to‐yellow/red spectral region, producing bright white light, as shown in the inset photograph. The corresponding CIE chromaticity coordinates fall near the white‐light region, confirming that Mn^4+^‐doped Li_2_HfF_6_ can contribute effectively to color tuning in white pc‐LEDs.

### 3.3 Cathodoluminescence and Evidence of STE2 → Mn^4+^ Energy Transfer

The CL spectra of the Li_2_HfF_6_ hosts without Mn are shown in Figure 6a. Gaussian deconvolution reveals two emission components, denoted STE1 and STE2. In Li_2_HfF_6_, STE1 is centered at 383.9 nm and accounts for 66.0% of the integrated emission area, whereas STE2 is centered at 432.3 nm and contributes 34.0%. In K‐doped Li_2_HfF_6_, the STE1 position remains similar at 387.5 nm, but its contribution decreases to 60.7%. By contrast, STE2 shifts to 448.1 nm, and its contribution increases to 39.3%. These results indicate that K incorporation redshifts the STE2 emission and increases its relative contribution to the CL spectrum.

**FIGURE 6:**
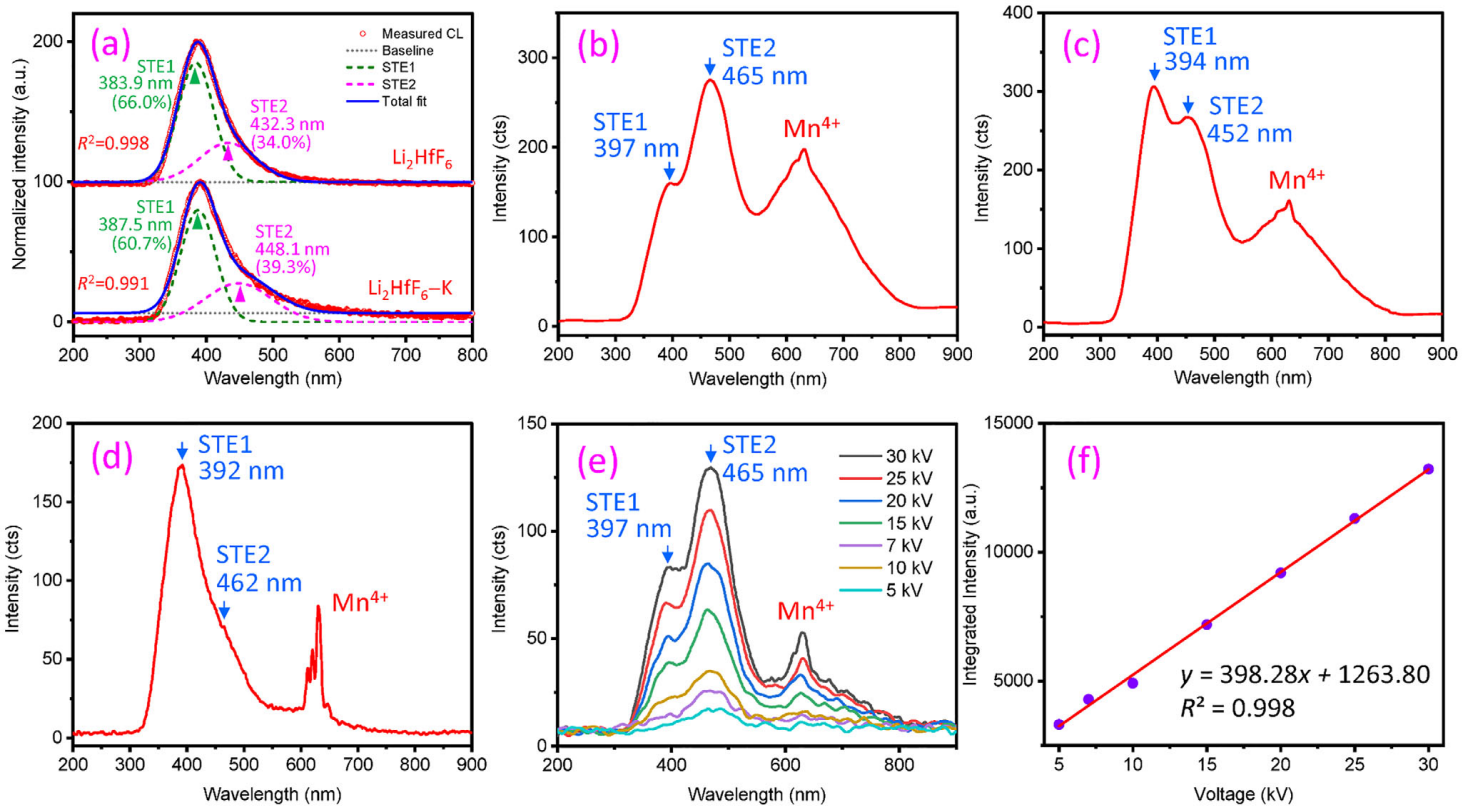
Cathodoluminescence. (a) Normalized CL spectrum of nominally undoped Li_2_HfF_6_ (upper) and K‐doped Li_2_HfF_6_ (bottom), with STE1 and STE2 contributions resolved by two‐component deconvolution. (b–d) Representative CL spectra of 2% Mn^4+^‐doped Li_2_HfF_6_ showing the correlation between STE2 and Mn^4^^+^ red emission. The spectra were collected from different microscopic regions of the same sample, yielding different relative contributions from STE1, STE2, and Mn^4+^ because of local compositional and structural inhomogeneity. (e) CL spectra were acquired at accelerating voltages from 5 to 30 kV with a constant current of 10 nA. (f) Integrated CL intensity vs. accelerating voltage.

Unlike conventional PL, which generally averages the emission over a comparatively large optically excited region, CL is generated within a localized electron–matter interaction volume and is therefore more sensitive to local structural and compositional variations. As shown in Figure 6b–d, the 2% Mn^4+^‐doped Li_2_HfF_6_ sample exhibits a strong correlation between the Mn^4+^ red emission and the relative intensities of STE1 and STE2. When STE2 is intense, the Mn^4+^ emission is also strong (Figure 6b). As the relative contribution of STE1 increases, the Mn^4+^ emission becomes weaker (Figure 6c). When STE1 dominates and STE2 is weak, only weak Mn^4+^ emission is observed (Figure 6d). This correlation suggests that direct excitation of Mn^4+^ by the electron beam is not the dominant excitation pathway under these conditions. Instead, electron‐beam excitation primarily generates electronic excitations in the host lattice, leading to the formation of STE1 and STE2, with STE2 serving as the principal sensitization channel for energy transfer to Mn^4+^. The different spectral weightings observed among the microscopic regions arise primarily from spatial variations in the relative STE1 and STE2 populations associated with inhomogeneous K distribution. Because STE2 sensitizes Mn^4+^ more efficiently, the relative Mn^4+^ emission contribution increases in STE2‐rich regions.

There are some differences in the STE emission ranges observed by PL and CL. In PL, the STE1 peak positions span approximately 312–416 nm, whereas the STE2 peaks occur within 405–467 nm. In CL, the corresponding ranges are narrower, with STE1 located at 384–397 nm and STE2 at 432–465 nm. Similar PL–CL shifts in STE emission have also been reported for Cs_2_HfF_6_ [19]. Generally, distinct luminescence responses under optical excitation and high‐energy radiation have been observed in a variety of phosphors [48, 49, 50, 51]. These differences can arise from the distinct excitation and sampling mechanisms of PL and CL. PL selectively excites optically accessible states, which may be influenced by surface defects and excitation wavelength, leading to a larger STE1 range. In CL, energetic electrons generate secondary electrons and electron–hole pairs throughout the interaction volume, probing deeper into the particles and minimizing the role of the surface [52], resulting in a smaller STE1 range.

A series of CL spectra were recorded at different accelerating voltages (Figure 6e), and the integrated area under the emission spectra is shown in Figure 6f as a function of voltage, showing a linear relationship. As the accelerating voltage *V* increases from 5 to 30 kV, the integrated CL intensity *I*_CL_ increases monotonically, and a linear fit gives

(4) $$I_{\mathrm{CL}}=398.28V+1263.80,\;\mathrm{with}\;R^{2}=0.998.$$

This near‐linear behavior indicates that the material responds predictably to increasing electron‐beam energy deposition in this range, an important requirement for radiation‐detection applications.

### 3.4 Self‐Trapped Exciton Modeling

Previous first‐principles studies by Chuklina and Mysovsky on self‐trapped holes in alkaline‐earth fluorides employed Hubbard corrections to the F 2p states [53]. Their parameter tests indicated that stable hole localization could be obtained within 6 ≤ *U* ≤ 13 eV and 1 ≤ *J* ≤ 4 eV. In another DFT + *U* study of self‐trapped holes in silver halides, Hubbard corrections were applied directly to the halogen p orbitals, with *U*_Cl(p)_ =  5 eV for AgCl and *U*_Br(p)_ =  3 eV for AgBr [54]. In the present study of Li_2_HfF_6_, we found that *U*_eff_ = 12 eV was required for fluorine to stabilize the hole localization.

We used a periodic 2 × 2 × 3 supercell containing 108 atoms. Trace Na impurities may also perturb local Li/F coordination because Na^+^ has a larger ionic radius than Li^+^. Such perturbations could contribute to the STE emission. Because K correlates more directly with the Mn precursor chemistry and produces a larger local size mismatch, the present DFT analysis focuses only on K substitution. For the K‐doped model, we replaced only one Li atom (#17 Li, denoted as Li17) with K for simplicity, corresponding to a 4.17% K substitution level. The DOS and band structures of the ground‐state pure Li_2_HfF_6_ and K‐doped Li_2_HfF_6_ models are shown in Figures S6a and S6c, respectively. Nominally undoped Li_2_HfF_6_ is a wide‐band‐gap fluoride host, with a calculated bandgap of 8.24 eV, whereas K incorporation slightly increases the bandgap to 8.42 eV. The valence‐band maximum is dominated by F 2p states, while the conduction‐band minimum is dominated by Hf 5d states. Their spin density distributions are uniform (Figure S6b,d).

To model the STE structure, A contracted F–F dimer (#61 and #65 fluorine atoms, denoted as F61 and F65, respectively) was introduced as the initial hole‐localization center, while a nearby Hf site (#11 hafnium atom, denoted as Hf11) was selected as the initial electron‐localization center. The spin occupations were manually specified on these selected atoms to initialize the localized STE configurations by full relaxation. In the ΔSCF calculations of the excited state, an electron was promoted from an occupied valence‐band state to an unoccupied conduction‐band state, and the atomic coordinates were relaxed while maintaining the excited‐state occupation [55, 56, 57, 58, 59, 60].

The DOS and spin‐resolved band structure of the relaxed intrinsic STE1 model are shown in Figure 7a, where the excited electron occupies an Hf11‐derived in‐gap state located approximately 0.38 eV below the calculated conduction‐band minimum, while the hole is associated with an F65‐derived state near the valence‐band edge. Its excitation‐induced charge‐density difference and spin density, calculated by ΔSCF, are shown in Figure 7b. The charge‐density difference, Δ*ρ*(**r**), is obtained as

(5) $$\Delta \rho \left(r\right)=\rho_{\mathrm{exc}}\;\left(r\right)-\rho_{\mathrm{GS}}\left(r\right),$$

where *ρ*_exc_(**r**) and *ρ*_GS_(**r**) are the charge densities of the excited and ground states, respectively. The charge‐density‐difference plot visually confirms electron accumulation around Hf11 and electron depletion at F65, consistent with the STE configuration (Figure 7b, left).

**FIGURE 7:**
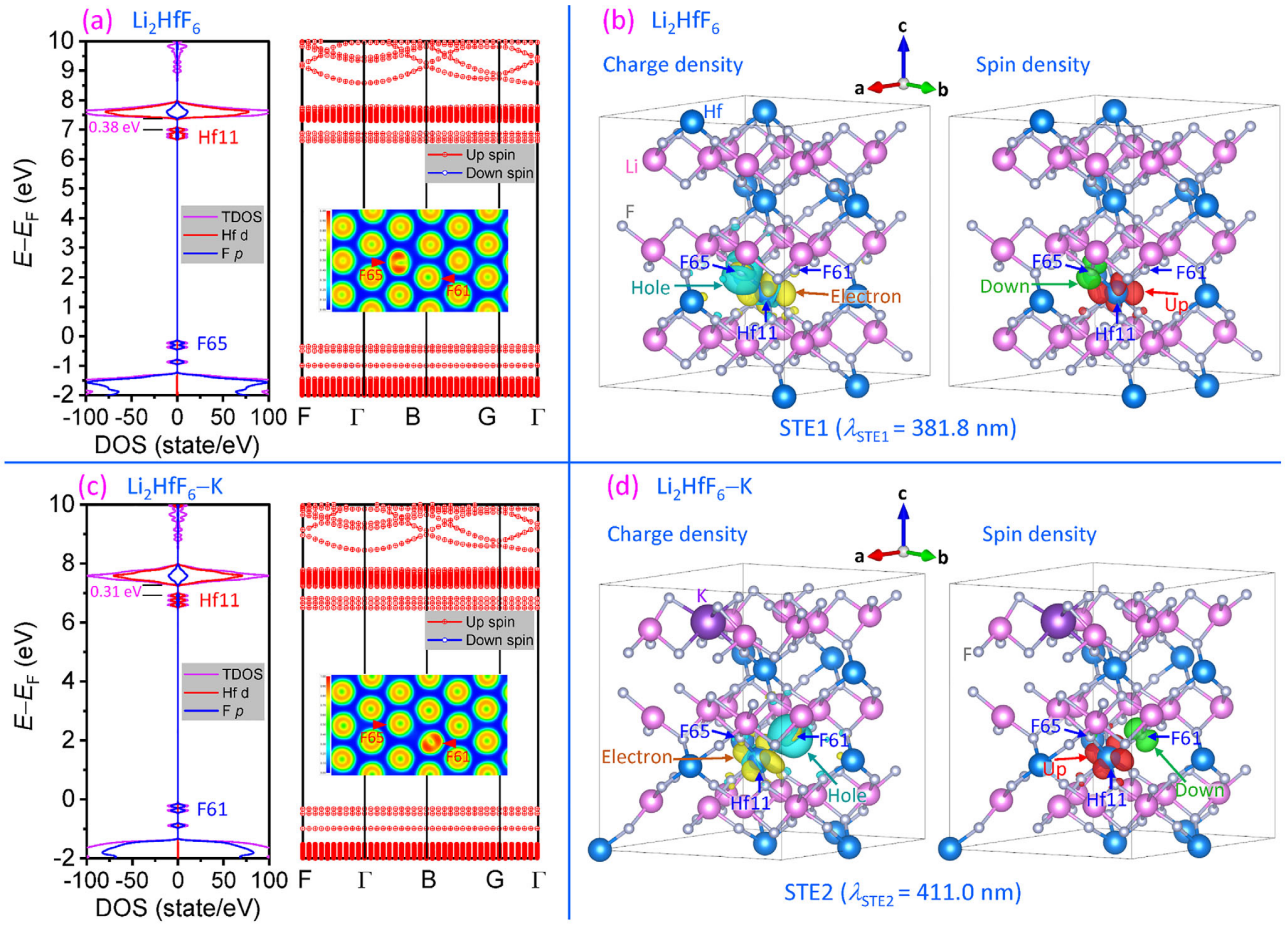
ΔSCF calculations of STEs. (a) DOS (left) and spin‐resolved band structure (right) of the relaxed intrinsic STE1 model in pure Li_2_HfF_6_. The inset shows the ELF calculated for the excited state in the (001) plane containing F65 and F61, with a pronounced high‐ELF region around F65. (b) Excitation‐induced charge‐density difference (left) and net spin density (right) for STE1, showing electron localization around Hf11 and hole localization at F65. The calculated STE1 emission wavelength is 381.8 nm. (c) DOS (left) and spin‐resolved band structure (right) of the relaxed K‐doped STE2 model. The inset shows the excited‐state ELF in the corresponding (001) plane, with a pronounced high‐ELF region around F61. (d) Excitation‐induced charge‐density difference (left) and net spin density (right) for STE2, showing electron localization around Hf11 and hole localization at F61. The calculated STE2 emission wavelength is 411.0 nm. Isosurfaces: yellow, electron accumulation; cyan, electron depletion; red, positive (up) spin density; green, negative (down) spin density.

The spin‐density distribution is defined as

(6) $$\rho_{\mathrm{spin}}\left(r\right)=\rho_{↑}\;\left(r\right)-\rho_{↓}\left(r\right),$$

where *ρ*_↑_(**r**) and *ρ*_↓_(**r**) are the spin‐up and spin‐down electron densities, respectively. Consistently, net spin density exhibits opposite polarizations at the electron‐ and hole‐localization sites (Figure 7b, right). The electron localization function (ELF) calculated for the excited state supports the localized character of the F65 p and Hf11 d states. An ELF map of the (001) plane containing F65 and F61 is shown in the inset of Figure 7a, revealing a pronounced high‐ELF region around F65. The position of this plane within the 3D crystal structure, together with ELF maps in the other (001) and (110) planes, is provided in Figure S7.

Using the configuration‐coordinate notation in Figure S8, the vertical emission energy of STE is given by [58, 60]

(7) $$E_{\mathrm{em}}=E_{\mathrm{abs}}\;-E_{\mathrm{ST}}-E_{\mathrm{deform}},$$

where *E*_abs_ is the vertical absorption energy from the ground‐state (GS) minimum to the free‐exciton (FE) state, *E*_ST_ is the self‐trapping energy, and *E*_deform_ is the lattice deformation energy, defined as the ground‐state energy cost of maintaining the STE‐distorted geometry. The self‐trapping energy is expressed as

(8) $$E_{\mathrm{ST}}=E_{\mathrm{abs}}\;-\left[E_{\mathrm{exc}}\left(R_{\mathrm{STE}}\right)-E_{\mathrm{GS}}\left(R_{\mathrm{GS}}\right)\right]=E_{\mathrm{exc}}\;\left(R_{\mathrm{GS}}\right)-E_{\mathrm{exc}}\left(R_{\mathrm{STE}}\right),$$

where *E*_exc_(*R*_STE_) is the excited‐state energy at the relaxed STE geometry *R*_STE_, and *E*_GS_(*R*_GS_) is the ground‐state energy at the equilibrium ground‐state geometry (*R*_GS_), and *E*_exc_(*R*_GS_) is the excited‐state energy at the GS geometry *R*_GS_. A positive *E*_ST_ (*E*_ST_ > 0) indicates that structural relaxation stabilizes the STE relative to the vertically excited free‐exciton configuration. The ground‐state deformation energy is

(9) $$E_{\mathrm{deform}}=E_{\mathrm{GS}}\left(R_{\mathrm{STE}}\right)-E_{\mathrm{GS}}\left(R_{\mathrm{GS}}\right),$$

where *E*_GS_(*R*_STE_) is the ground‐state energy evaluated at the STE‐distorted geometry. Equivalently, the vertical emission energy is

(10) $$E_{\mathrm{em}}=E_{\mathrm{exc}}\;\left(R_{\mathrm{STE}}\right)-E_{\mathrm{GS}}\left(R_{\mathrm{STE}}\right).$$

The calculated results are listed in Table 3. The calculated STE1 emission wavelength is 3.25 eV (381.8 nm), which falls within the 312–416 nm range in PL and near the 384–397 nm range in CL.

**TABLE 3:** ΔSCF calculation of energy components for STE1 and STE2.

Model	*E*_abs_ (eV)	*E*_em_ (eV)	*E*_deform_ (eV)	*E*_ST_ (eV)
STE1 (Li_2_HfF_6_)	6.45	3.25 (381.8 nm)	0.84	2.36
STE2 (Li_2_HfF_6_−K)	6.31	3.02 (411.0 nm)	1.54	1.75

The DOS and band structure of the K‐doped STE2 model are shown in Figure 7c. As in STE1, the excited electron occupies an Hf11‐derived localized state near the conduction‐band edge. However, K incorporation perturbs the local Li/F coordination environment and shifts the hole localization from F65 to F61. The occupied Hf11‐derived state lies approximately 0.31 eV below the calculated conduction‐band minimum, which is slightly closer to the band edge in STE1. The charge‐density‐difference and spin‐density maps further support electron localization near Hf11 and hole localization on F61 (Figure 7d). The excited‐state ELF map of the (001) plane containing F61 and F65 is shown in the inset of Figure 7c, where a pronounced high‐ELF region around F61 is clearly visible.

The ΔSCF calculation yields an STE2 emission energy of 3.02 eV, corresponding to 411.0 nm (Table 3), a redshift of 0.23 eV relative to the calculated STE1. This value lies within the broad excitation‐dependent PL range of approximately 405–467 nm and is close to the CL band of 432–465 nm. These results demonstrate that K impurities at Li sites generate a distinct, redshifted, lower‐energy STE center.

Because the two structural variants associated with STE1 and STE2 coexist within the sample, the measured PL and CL spectra may include contributions from both local environments. We assign STE1 and STE2 based on combined evidence from their distinct spectral ranges, consistent PL and CL behavior, and corresponding DFT‐calculated emission energies. Some spectral averaging between the coexisting phases may occur. Nevertheless, the agreement among these independent experimental and computational results supports the STE1 and STE2 assignments.

## 4 Conclusions

In summary, Li_2_HfF_6_ exhibits an unusual impurity‐associated phase separation that gives rise to two distinct self‐trapped exciton states: intrinsic STE1 and a redshifted, K‐associated STE2, as consistently supported by PL, CL, and first‐principles calculations. Under high‐energy CL excitation, strong Mn^4+^ red emission is observed only in regions where STE2 is prominent, supporting a selective STE2 → Mn^4+^ sensitization pathway. Although K is nonuniformly distributed, its incorporation beneficially modifies the local lattice and stabilizes STE2, thereby promoting host‐to‐activator energy transfer. These results identify nominal alkali‐metal impurities as active regulators of excitonic states rather than passive residual species and demonstrate impurity modulation as an effective strategy for tuning STE emission and activator sensitization in complex fluoride luminescent materials.

## Author Contributions

M. Adhikari conducted sample synthesis, XRD, SEM, and part of the PL measurements. T. Murray, C. Lewis, and J. Wu conducted the sample synthesis. H. Adhikari and J. Wen conducted TEM. C. Li and H. Wu conducted NPD and refinement. Z. Luo designed and supervised the experiment, conducted CL, part of PL, and DFT, and prepared the paper.

## Conflicts of Interest

The authors declare no conflicts of interest.

## Supporting information




**Supporting File**: advs78338‐sup‐0001‐SuppMat.pdf.

## Data Availability

The data that supports the findings of this study are available in the supplementary material of this article.
