# Supplementary material for: Potassium‐Impurity‐Modulated Self‐Trapped Excitons and Energy Transfer in Phase‐Separated, Mn4+‐Doped Li2HfF6
Source: Adv Sci (Weinh). 2026 Oct 27:e78338. Online ahead of print. doi: 10.1002/advs.78338 (PMC13712481; doi:10.1002/advs.78338)
Supplement: Supplementary file 1 [file ADVS-9999-e78338-s001.pdf]

Potassium-Impurity-Modulated Self-Trapped Excitons and Energy Transfer in Phase-Separated, Mn^{4+}-doped Li_2HfF_6

Menuka Adhikari[1], Thomas Murray[1], Cameron J. Lewis[1], Jett Wu[1], Hari Adhikari[2], Cheng Li[3], Jianguo Wen[2], Hui Wu[4], and Zhiping Luo[1, *]

[1]Department of Chemistry, Physics, and Materials Science, Fayetteville State University, Fayetteville, North Carolina 28301, USA
[2]Center for Nanoscale Materials, Argonne National Laboratory, Lemont, Illinois 60439, USA
[3]Neutron Scattering Division, Oak Ridge National Laboratory, Oak Ridge, Tennessee 37830, USA
[4]NIST Center for Neutron Research, National Institute of Standards and Technology, Gaithersburg, Maryland 20899, USA

* Corresponding author. E-mail: zluo@uncfsu.edu

Table S1. DFT-calculated lattice expansion induced by replacing one Li atom with K in Li_2HfF_6 supercells. The unit cell was first fully relaxed, yielding lattice parameters of a = 4.848 Å and c = 4.534 Å. Pure supercells were then constructed from the relaxed unit cell and fully relaxed. For each K-substituted model, one Li atom was replaced by K, followed by full relaxation of the atomic coordinates and lattice parameters. The effective unit-cell parameters a and c were obtained by dividing the relaxed supercell dimensions by the corresponding replication factors. The relative lattice expansions were calculated with respect to the corresponding relaxed pure supercells.

Supercell	Composition	K substitution level (%)	$a_{\text{supercell}}$ (Å)	$c_{\text{supercell}}$ (Å)	Effective a (Å)	Effective c (Å)	Expansion along a	Expansion along c
2×2×2	Pure		9.707	9.078	4.854	4.539		
	K-doped	6.25% (1/16)	9.853	9.214	4.927	4.607	1.50%	1.50%
2×2×4	Pure		9.707	18.155	4.853	4.539		
	K-doped	3.13% (1/32)	9.783	18.296	4.891	4.574	0.78%	0.78%
2×2×5	Pure		9.698	22.673	4.849	4.535		
	K-doped	2.50% (1/40)	9.756	22.808	4.878	4.562	0.59%	0.59%
2×2×6	Pure		9.698	27.208	4.849	4.535		
	K-doped	2.08% (1/48)	9.747	27.343	4.873	4.557	0.50%	0.50%
3×3×4	Pure		14.549	18.140	4.850	4.535		
	K-doped	1.39% (1/72)	14.597	18.200	4.866	4.550	0.33%	0.33%

Table S2. PL decay fitting parameters from five measurements under 260 and 467 nm excitation.

No.	y_0	A_1	τ_1 (ms)	A_2	τ_2 (ms)	τ_{ave} (ms)	Adj.-R^2
**Excitation: 260 nm**							
1	0.00142	0.32151	1.03222	0.65059	4.18975	3.85	0.99984
2	0.00136	0.32231	1.12358	0.65404	4.24954	3.89	0.99987
3	0.00144	0.31678	0.94871	0.64124	4.13161	3.81	0.99979
4	0.00146	0.31892	0.95002	0.64463	4.13078	3.81	0.99979
5	0.00145	0.31886	0.99143	0.64964	4.15985	3.83	0.99982
**Average**	**0.00143**	**0.31968**	**1.00919**	**0.64803**	**4.17231**	**3.84**	**0.99982**
**Std. dev.**	**0.00004**	**0.00223**	**0.07261**	**0.00507**	**0.04951**	**0.03**	**0.00003**
**Excitation: 467 nm**							
1	0.00114	0.32056	0.89589	0.60122	3.96948	3.64	0.9997
2	0.00114	0.32709	0.90233	0.60910	3.97178	3.64	0.99972
3	0.00112	0.33538	0.94005	0.61462	4.00350	3.66	0.99976
4	0.00117	0.32088	0.88188	0.59932	3.95698	3.63	0.99967
5	0.00114	0.32104	0.88376	0.59786	3.96132	3.63	0.99968
**Average**	**0.00114**	**0.32499**	**0.90078**	**0.60442**	**3.97261**	**3.64**	**0.99971**
**Std. dev.**	**0.00002**	**0.00641**	**0.02353**	**0.00716**	**0.01828**	**0.01**	**0.00004**

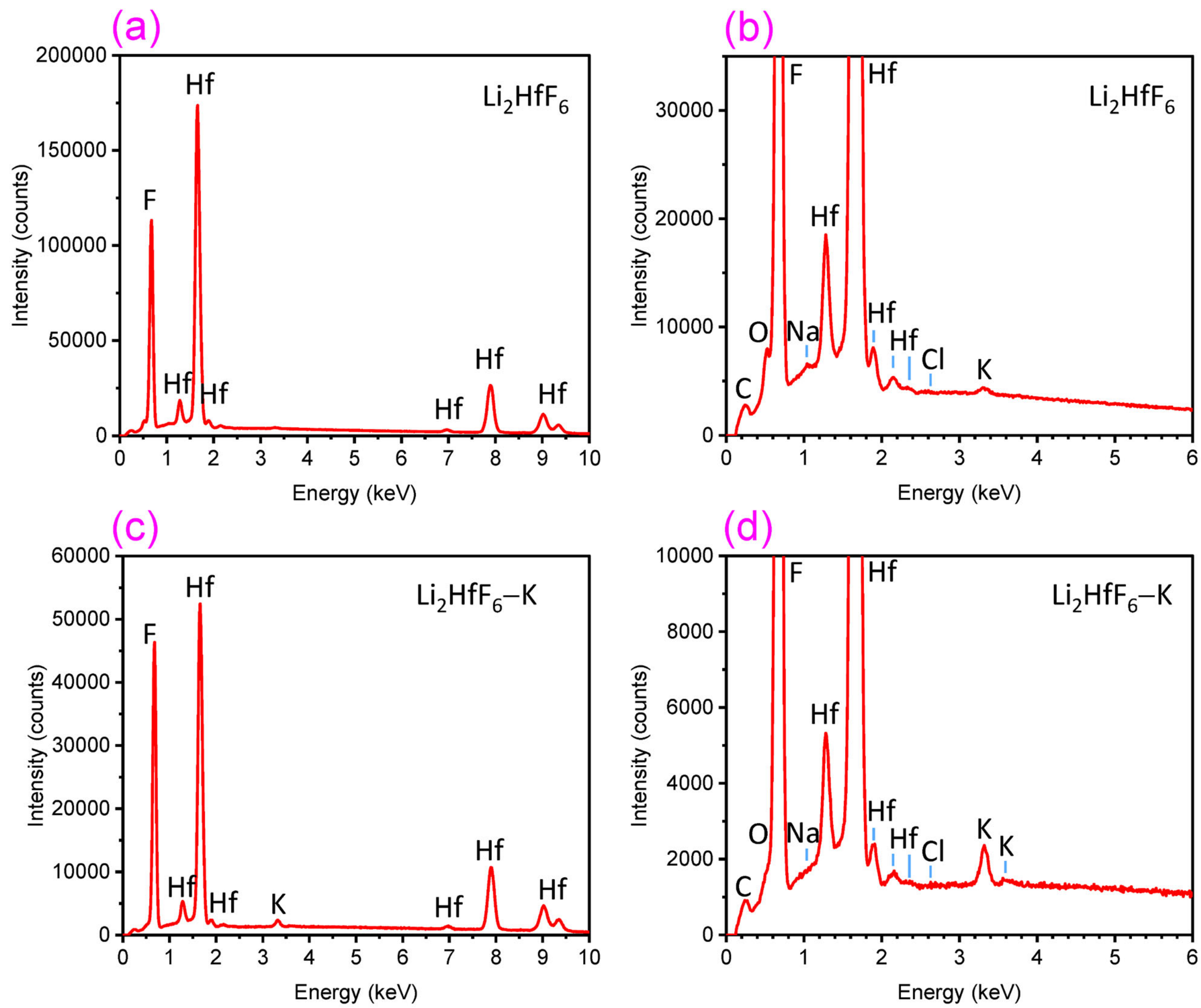

Figure S1. EDS spectra of nominally undoped Li_2HfF_6 acquired for 3 min: (a) full spectrum and (b) enlarged low-energy region showing trace Na and K. (c, d) Corresponding spectra of intentionally K-containing Li_2HfF_6, showing an enhanced K peak. C and O are attributed primarily to surface or electron-beam-induced contamination. The expected Cl peak position is marked, but no resolved Cl peak is observed in (b) and (d). Na is also not resolved in (d). Li is not detectable by EDS.

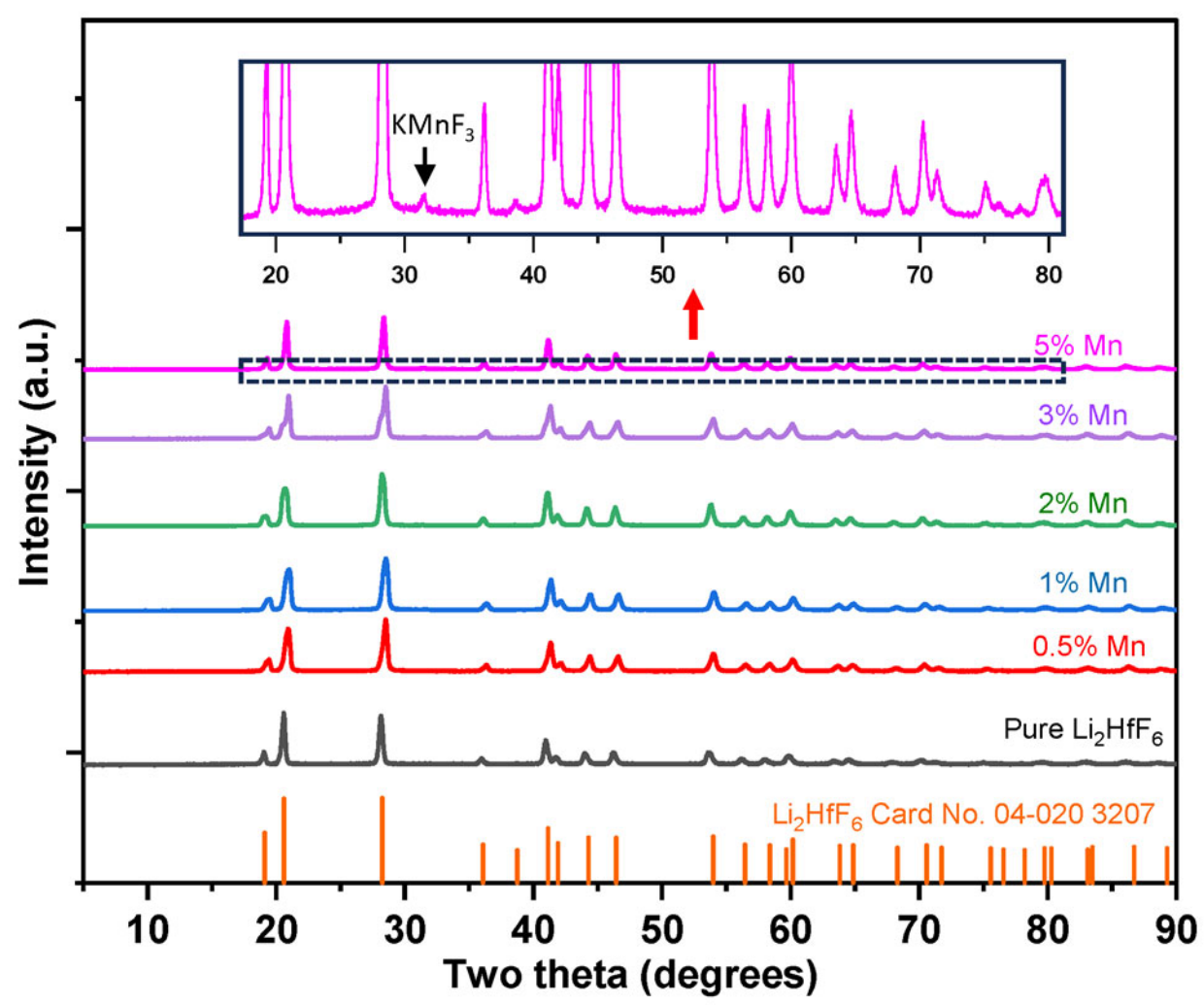

Figure S2. Powder XRD patterns of Li_2HfF_6 containing 0 (undoped), 0.5, 1, 2, 3, and 5% Mn, compared with the reference Li_2HfF_6 pattern (card No. 04-020-3207). Samples up to 3 % Mn retain the host pattern, whereas the 5% Mn sample shows a very small peak from $KMnF_3$, as shown in the inset at the top.

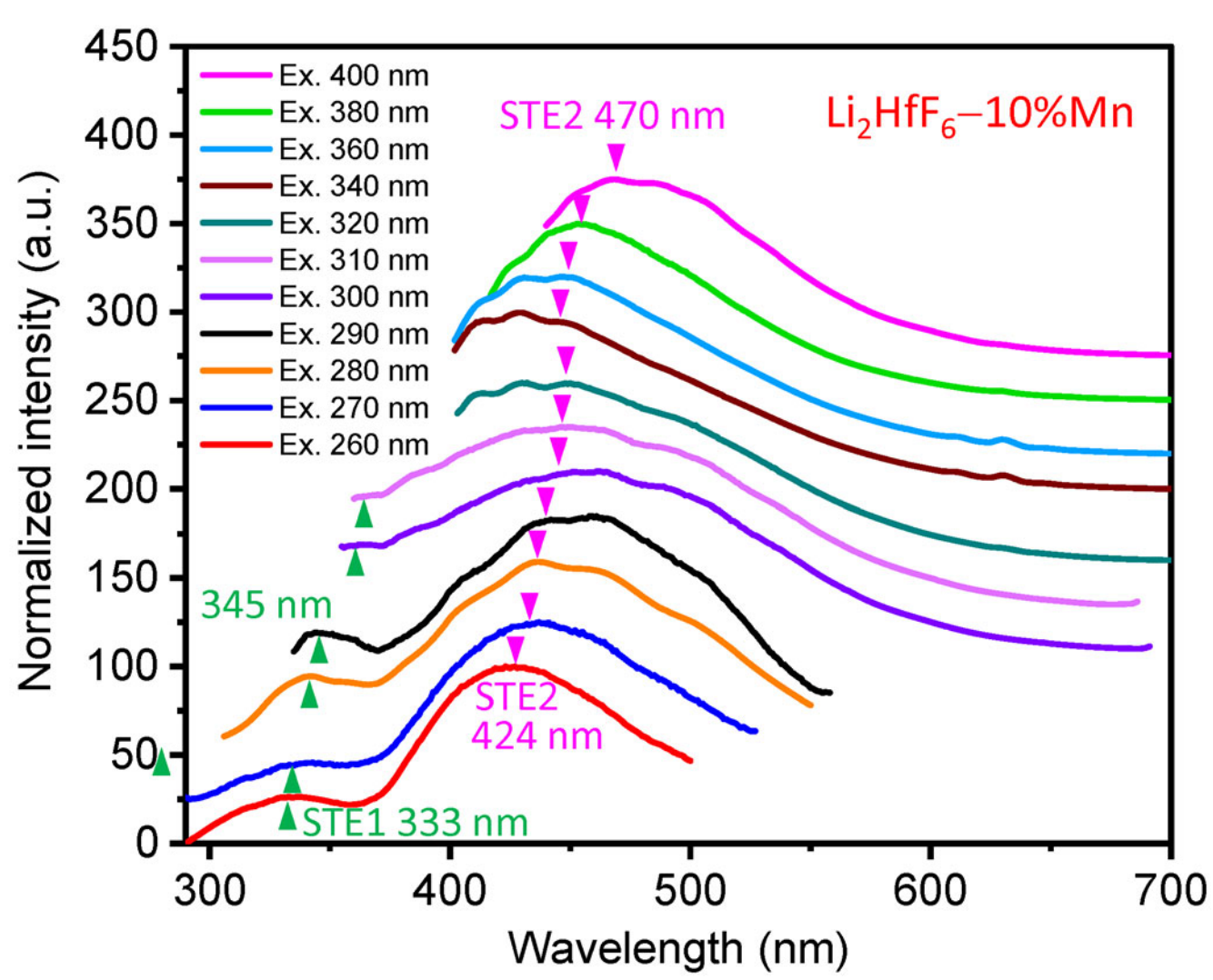

Figure S3. Excitation-dependent PL spectra of Li_2HfF_6 containing 10% Mn, recorded from 260 to 400 nm excitation. The labels identify the higher-energy STE1 contribution and the excitation-dependent lower-energy STE2 component.

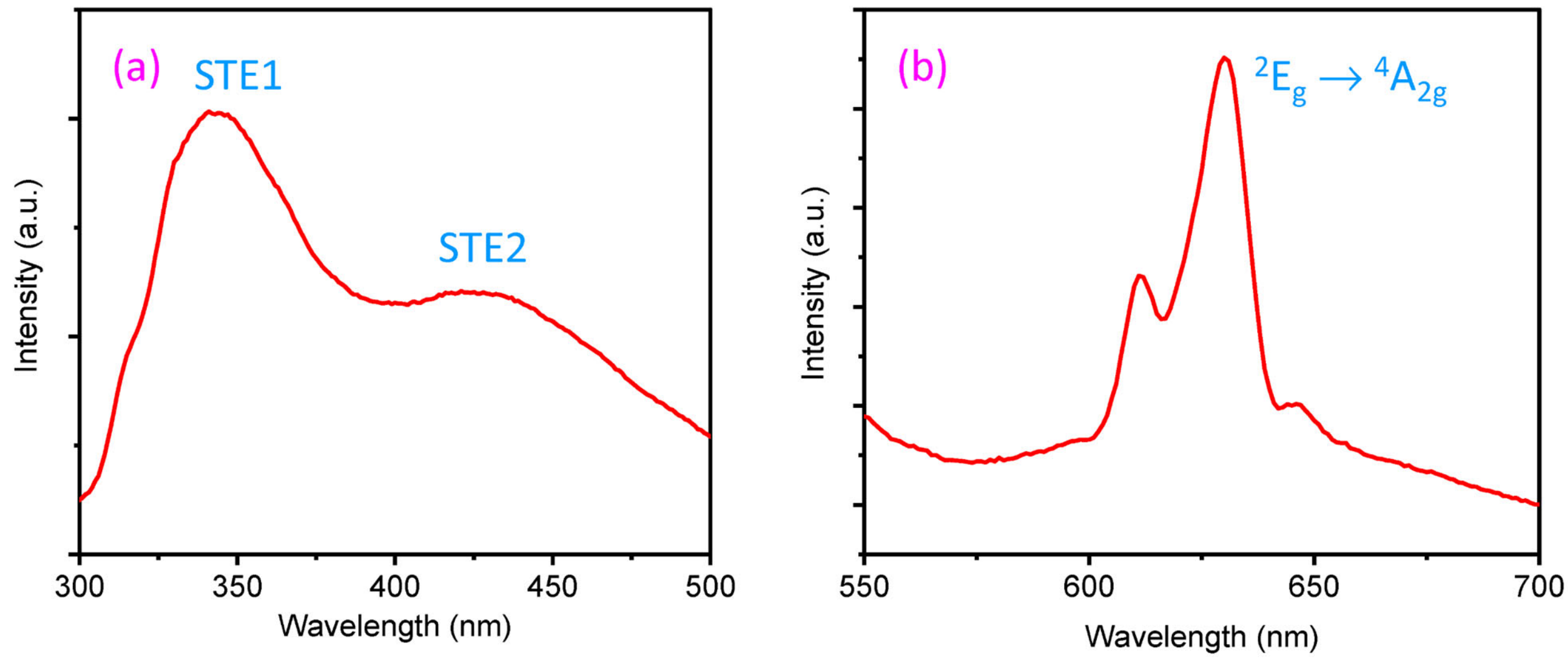

Figure S4. PL emission spectra under 260 nm excitation. (a) Emission spectrum in the 300–500 nm range, showing the STE1 and STE2 emissions. (b) Emission spectrum in the 550–700 nm range, showing the characteristic Mn^{4+} red emission. The spectral region around 520 nm was excluded because of the second-order diffraction artifact from the 260 nm excitation wavelength.

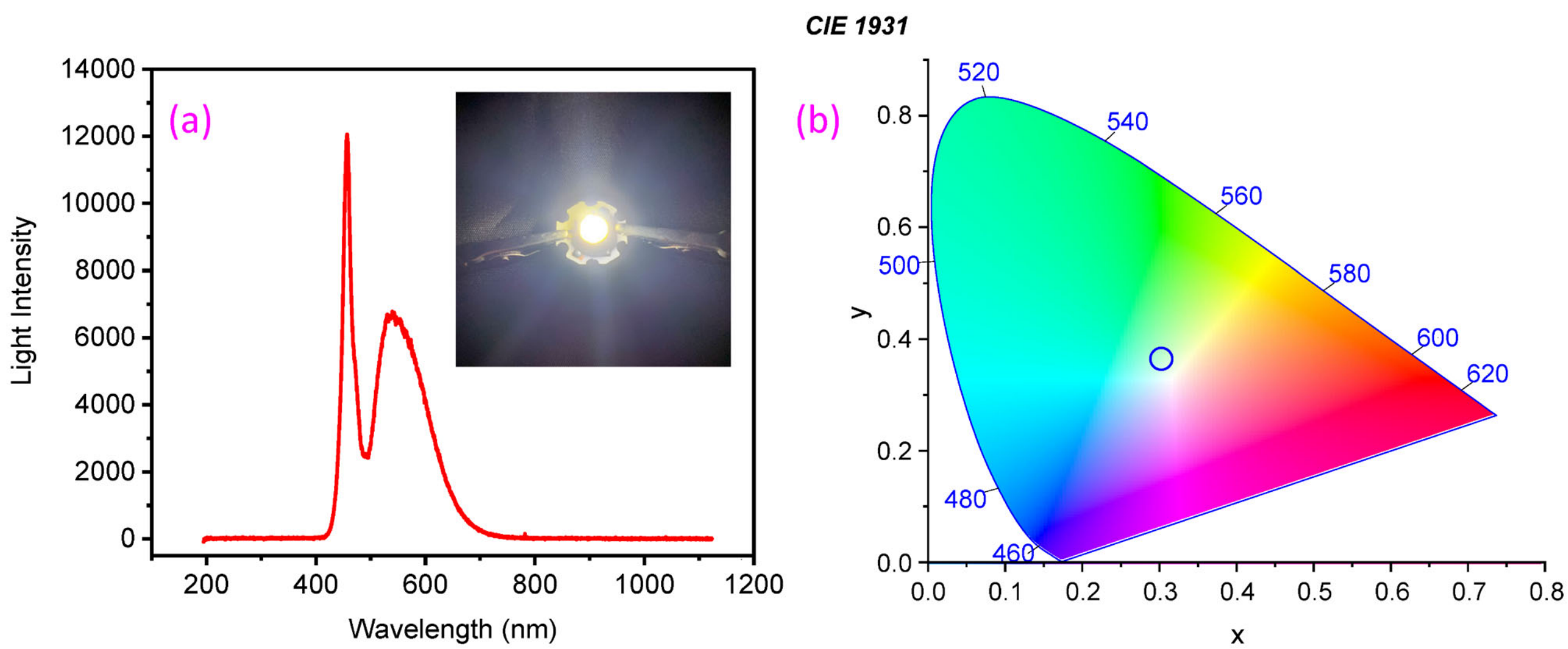

Figure S5. (a) Emission spectrum of the pc-LED, fabricated by coating Mn^{4+}-doped Li_2HfF_6 and yellow YAG phosphors onto a blue LED chip. The inset shows a photograph of the operating pc-LED, exhibiting bright white-light emission. (b) CIE 1931 chromaticity diagram showing the emission coordinates of the pc-LED located near the white-light region.

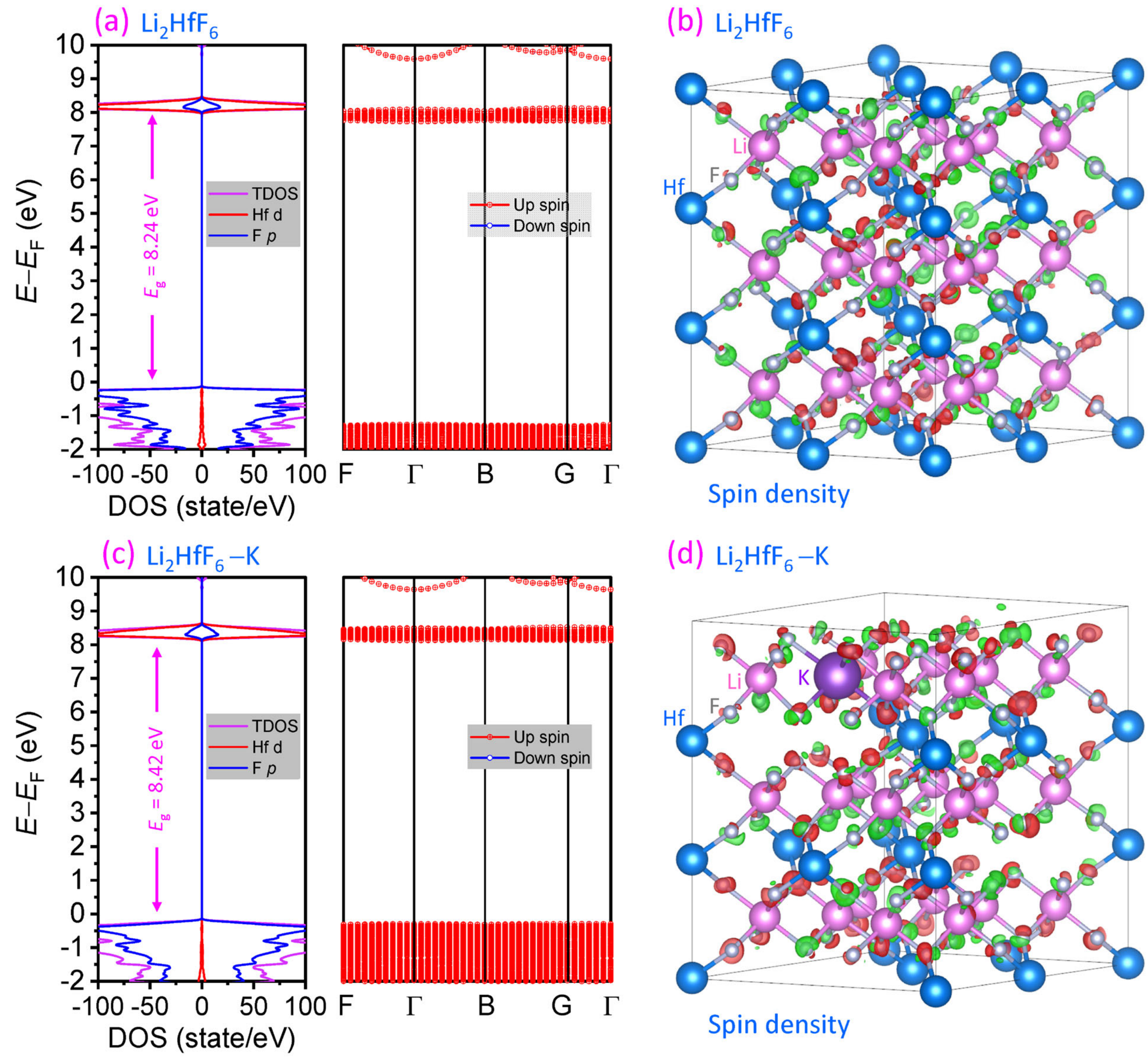

Figure S6. Ground-state DOS and spin-resolved band structures of (a) Li_2HfF_6 and (c) K-containing Li_2HfF_6, together with the corresponding spin densities in (b) and (d). The calculated gaps are 8.24 and 8.42 eV, respectively. Isosurfaces: red, positive (up) spin density; green, negative (down) spin density.

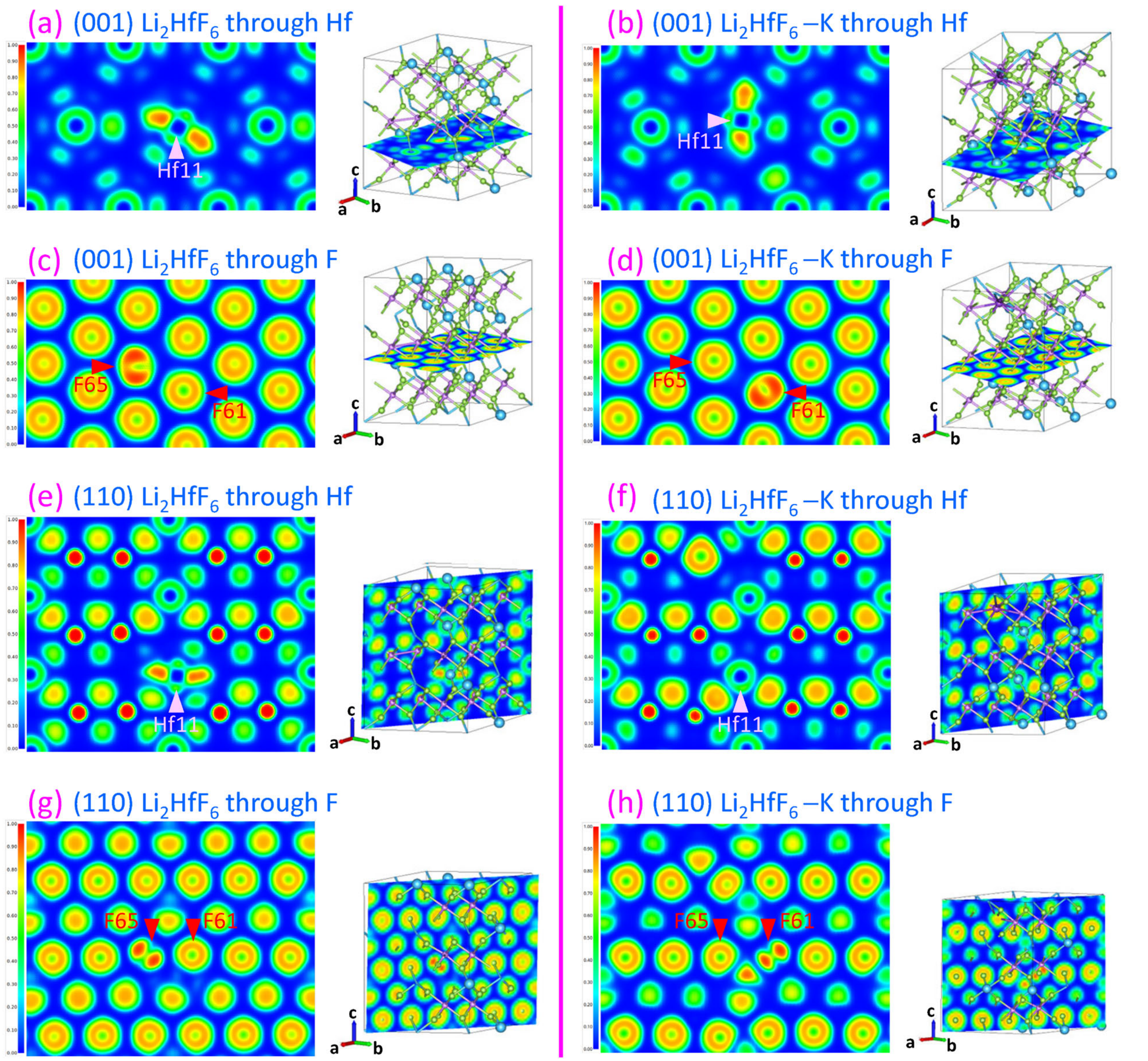

Figure S7. Electron localization function (ELF) maps at the excited state of pure Li_2HfF_6 (left column) and K-doped Li_2HfF_6 (right column). Maps are shown for the (001) planes passing through Hf (a, b) and F (c, d), and the (110) planes passing through Hf (e, f) and F (g, h). The corresponding locations of the selected planes within the three-dimensional crystal structures are shown to the right of each ELF map.

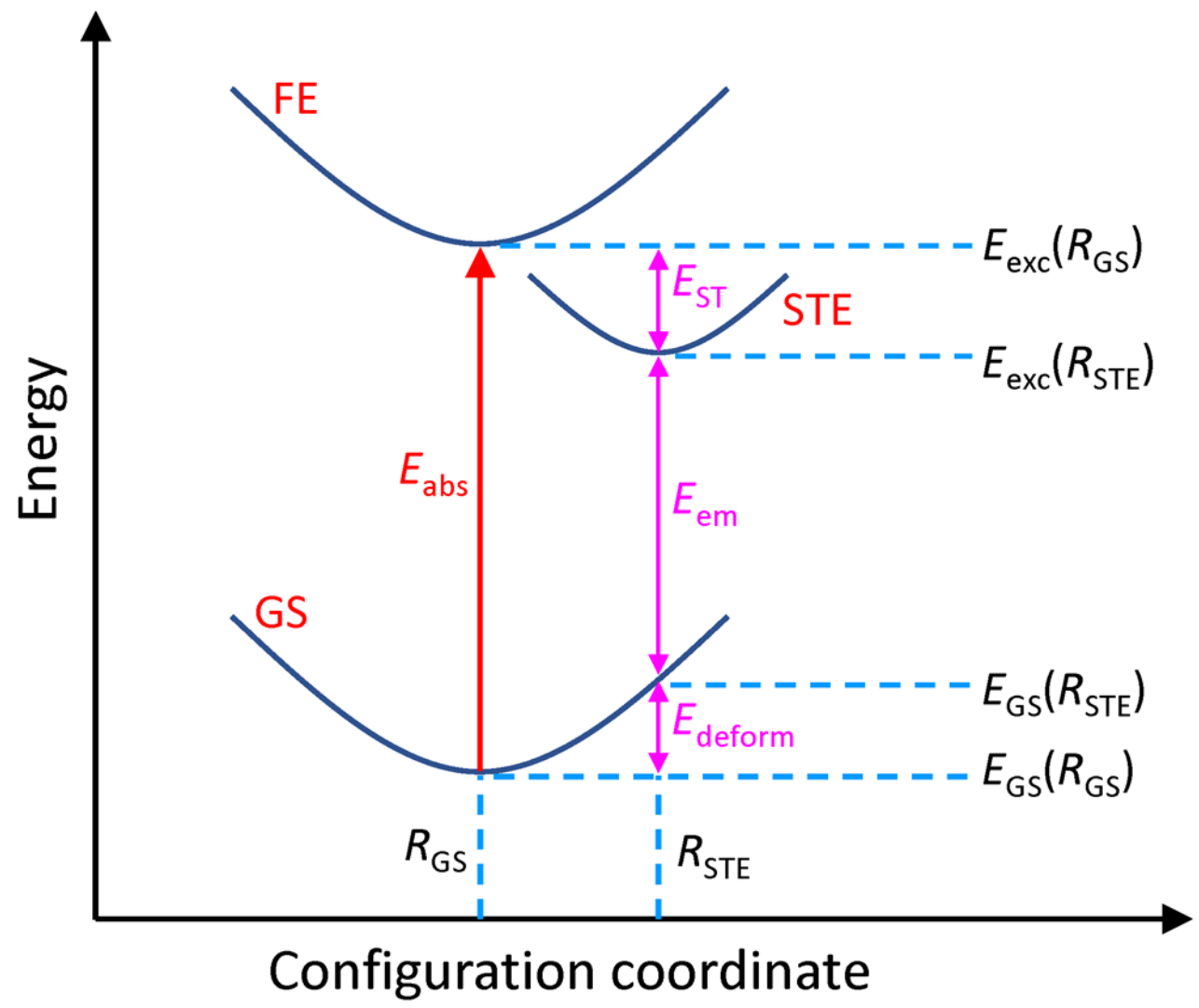

Figure S8. Schematic configuration-coordinate diagram showing the energy relationships used in the ΔSCF calculations. GS: ground-state potential-energy surface; FE: vertically excited/free-exciton-like configuration; STE: relaxed self-traped-exciton configuration; R_{GS}: ground-state geometry; R_{STE}: STE geometry; E_{abs}: vertical absorption energy; E_{ST}: self-trapping energy; E_{em}: vertical emission energy at the relaxed STE geometry; E_{deform}: ground-state energy cost of the STE-distorted geometry. These energies satisfy $E_{abs} = E_{ST} + E_{em} + E_{deform}$.
